# Highly Sensitive Detection of *IDH2* Mutations in Acute Myeloid Leukemia

**DOI:** 10.3390/jcm9010271

**Published:** 2020-01-19

**Authors:** Jessica Petiti, Valentina Rosso, Eleonora Croce, Vanessa Franceschi, Giacomo Andreani, Matteo Dragani, Marco De Gobbi, Monia Lunghi, Giuseppe Saglio, Carmen Fava, Marco Lo Iacono, Daniela Cilloni

**Affiliations:** 1 Department of Clinical and Biological Sciences of the University of Turin, San Luigi Gonzaga Hospital, Regione Gonzole 10, 10043 Orbassano (Turin), Italy; valentina.rosso@gmail.com (V.R.); eleonora.croce@edu.unito.it (E.C.); vanessa.franceschi93@gmail.com (V.F.); giacomo.andreani@unito.it (G.A.); matteo.dragani@gmail.com (M.D.); marco.degobbi@unito.it (M.D.G.); giuseppe.saglio@unito.it (G.S.); carmen.fava@unito.it (C.F.); marco.loiacono@unito.it (M.L.I.); 2 Division of Hematology, Department of Translational Medicine, Università del Piemonte Orientale, Corso Giuseppe Mazzini, 18, 28100 Novara, Italy; monia.lunghi@med.uniupo.it

**Keywords:** AML, IDH2 R140Q, IDH2 R172K, PNA-PCR clamping, droplet digital PCR, diagnostic assay

## Abstract

Background: Acute myeloid leukemia is a heterogeneous hematological disease, characterized by karyotypic and molecular alterations. Mutations in *IDH2* have a role in diagnosis and as a minimal residue disease marker. Often the variant allele frequency during follow up is less than 20%, which represents the limit of detection of Sanger sequencing. Therefore, the development of sensitive methodologies to identify *IDH2* mutations might help to monitor patients’ response to therapy. We compared three different methods to identify and monitor *IDH2* mutations in patients’ specimens. Methods: Performances of PNA-PCR clamping, droplet digital PCR and Sanger for *IDH2* status identification were evaluated and compared in 96 DNA patients’ specimens. Results: In contrast with Sanger sequencing, our results highlighted the concordance between PNA clamping and digital PCR. Furthermore, PNA-PCR clamping was able to detect more mutated DNA with respect to Sanger sequencing that showed several false negatives independently from the allelic frequency. Conclusions: We found that PNA-PCR clamping and digital PCR identified *IDH2* mutations in DNA samples with comparable results in a percentage significantly higher compared to Sanger sequencing. PNA-PCR clamping can be used even in laboratories not equipped for sophisticated analyses, decreasing cost and time for *IDH2* characterization.

## 1. Introduction

Acute myeloid leukemia (AML) is a malignant neoplasm characterized by a blockade in the differentiation of hematopoietic stem cells, with consequent abnormal accumulation of immature myeloid blasts and reduced production of mature blood cells [1]. AML is one of the most frequent disorders, constituting about 30% of cases of leukemia in adulthood [2]. Its incidence increased significantly with age, with an average at diagnosis of 65 years [3,4]. Although modern medicine tries to characterize AML, its prognostic stratification is still based on cytogenetic and on the detection of known mutations. The development of effective methodologies to identify new targets and to stratify patients is mandatory, with the goal of allowing each patient to receive a tailored therapy and to predict the probability of response. Although the precise prognostic significance of some molecular markers is still unclear, they might become interesting for the presence of specific drugs that target them: this is the case of the gene “isocitrate dehydrogenase 2” (*IDH2*) [5]. The Food and Drug Administration has recently approved Enasidenib, a specific molecular inhibitor for patients with AML with mutated *IDH2*. *IDH2* is an enzyme that catalyzes the first oxidative decarboxylation reaction of the isocitrate to α-ketoglutarate (α-KG) in the tricarboxylic acid cycle. Mutations in the *IDH2* gene occur in 8–19% of patients with AML [6], with high frequencies in older patients. The most frequent mutations of *IDH2*, which affect over 95% of *IDH2* mutated patients, involve the arginine residues in position R140 and R172. Mutant proteins acquire the ability to reduce the α-KG to (R)-2-hydroxyglutarate (2-HG). This oncometabolite competitively inhibits α-KG-dependent epigenetic regulators, including histone demethylases. Consequently, 2-HG accumulation leads to DNA hypermethylation, blocking cellular differentiation [7,8,9]. Furthermore, 2-HG also involves RNA epigenetic modification, especially N6-methyladenosine (m6A), via FTO [10]. The persistence of *IDH2* mutations was observed in about 40% of AML patients in complete remission (CR) or in complete remission with incomplete hematologic recovery (CRi) and is associated with a greater risk of recurrence [11]. This suggested the use of *IDH2* as possible molecular markers for MRD, particularly in the absence of other molecular alterations [12]. For these reasons, it is mandatory to monitor *IDH2* mutations to better characterize AML patients. To evaluate the *IDH2* status in AML patients, Sanger sequencing and droplet digital PCR (ddPCR) are considered useful molecular approaches. Sanger sequencing is the most used method with a limitation due to its poor limit of detection (−20%), in contrast, ddPCR has recently emerged as a highly sensitive and accurate technology [13]. Both these methods have the disadvantage of needing expensive apparatus and reagents. With the purpose of identifying a new molecular technique that is fast and cheap but with a sensitivity comparable to ddPCR, we developed a novel assay using peptide nucleic acid (PNA)-PCR clamping to detect *IDH2* R140Q and R172K mutations. PNA is a synthetic polymer analogous to DNA and RNA, with a skeleton characterized by repeating N-(2-aminoethyl)-glycine units linked by peptide bonds [14]. Unlike primers, PNA probes lack pentose sugar-phosphate groups, so PNA/DNA binding is stronger than DNA/DNA duplex. In addition, PNA/DNA complex is so specific that a single base mismatch can destabilize it [15]. Finally, PNA oligomers are not degraded or recognized by polymerase and therefore cannot be directly used as primers [16]. Our method exploits the ability of PNA to hybridize very specifically to DNA, without being extended by a polymerase, consequently suppressing DNA amplification [17,18,19].

## 2. Experimental Section

### 2.1. Patient’s Cohort

After informed consent, 96 DNA was extracted from human bone marrow or peripheral blood of AML patients (74 at diagnosis and 22 during follow up). DNA was extracted using Maxwell 16 Blood DNA Purification kit (Promega, Milan, Italy), following the manufacturer’s instructions. Patients were characterized at the cytogenetic level by conventional karyotyping and screened by Real-Time PCR for the presence of the most frequent fusion transcripts, as previously described [20]. *NPM1* [21] and *FLT3* ITD [22] mutations were screened and *WT1* mRNA levels were also evaluated [23]. Patients younger than 60 years were treated following standard protocols established by the GIMEMA Cooperative Group for the treatment of adult patients with AML [17]. Elderly and unfit patients were treated as previously described [17]. The study was approved by the local ethics committee of San Luigi Hospital, Orbassano, Turin (Number of approval 201/2014).

### 2.2. Cloning PCR Controls with pGEM^®^—T Easy Vector

Plasmids used as positive controls were generated amplifying *IDH2* R140Q and R172K from mutated AML patients with the following primers: forward 5′-AGACTCCAGAGCCCACACAT-3′ and reverse 5′-CTCGTCGGTGTTGTACATGC-3′. Subsequently, the PCRs were purified by QIAquick Gel Extraction Kit (Qiagen, Hildem, Germany) and cloned in pGEM-T Easy Vector (Promega, Milan, Italy). The sequences were verified by the capillary Sanger sequence method. All reactions were performed following the manufacturer’s instructions.

### 2.3. Sanger Sequencing for IDH2mut Detection

To perform Sanger sequencing, *IDH2* was amplified from DNA (50 ng) of AML patients and analyzed by sequencing with BigDye terminator v3.1 (Applied Biosystem, Foster City, CA, USA) and capillary electrophoresis on ABI PRISM 3130XL (Applied Biosystem, Foster City, CA, USA), using primers described by Marcucci et al. [24]. Out of 96 patients’ samples, 91 were efficiently sequenced, whereas for 5 samples, the sequences failed probably due to the quality of DNA. The sensitivity of the method was previously estimated by serial dilutions experiments to be approximately 15–20% [25].

### 2.4. ddPCR for IDH2mut Detection

Detection of *IDH2* R140Q and R172K (#dHsaMDV2010057 and #dHsaMDV2010059, respectively, Biorad, Hercules, CA, USA) was performed by QX200 ddPCR system (Biorad, Hercules, CA, USA). For *IDH2mut* detection, 100 ng of each patient’s DNA and 0.2 pg of control plasmids were used. All reactions were performed following the manufacturer’s instructions and generated evaluable data. Results were expressed as a percentage of mutated allele compared to wild type (WT) [(IDH2mut/IDH2) ×100].

### 2.5. PNA Directed PCR Clamping for IDH2mut Detection

The method for the detection of *IDH2* mutations by PNA-PCR clamping forecasted two PCR to detect IDH2 R140Q and R172K, respectively. Primers and PNA probes for *IDH2* amplification were designed on DNA sequences NG_023302.1. DNA of patients (250 ng) were used to amplify a small area of *IDH2*. PCR amplification for each mutation was carried out in duplicate in the presence (PNA+) or in the absence (PNA−) of PNA probes. Primers and PNA probes sequences are listed in Table 1.

PNA-PCR clamping for *IDH2* R140Q: reaction volume was 50 μL and the final concentrations of the reagents were as follow: MgCl_2_ [2.5 μM], 10X PCR Buffer [1X], dNTPs [200 nM], AmpliTaq 1U, Primers [100 nM each], PNA probe [300 nM]. PCR conditions were as follows: 95 °C 2 min, (95 °C 15 s, 55 °C 15 s, 68 °C 30 s) for 40 cycles, 68 °C 5 min. PNA PCR clamping for *IDH2* R172K: reaction volume was 50 μL and the final concentrations of the reagents were as follows: MgCl_2_ [2.5 μM], 10× PCR Buffer [1×], dNTPs [200 nM], AmpliTaq 1U, Primers [200 nM each], PNA probe [800 nM]. PCR conditions were as follows: 95 °C 2 min, (95 °C 30 s, 58 °C 20 s, 68 °C 30 s) for 40 cycles, 68 °C 5 min. 1 pg of each plasmid was used as control in PCR amplification. After PCR, 10 μL of each amplicon were loaded on 2% Agarose-TBE 1× gel with 5 μg/mL Ethidium Bromide (EtBr) and run at 120 V for 30 min. The electrophoretic runs were acquired with the ChemiDoc XRS+ (Biorad, Hercules, CA, USA) and analyzed with the Image Lab software 4.0.1 (Biorad, Hercules, CA, USA). All reactions for each *IDH2* mutation generated evaluable data that was compared to the other methodologies.

### 2.6. Statistical Analysis

Diagnostic test equivalency was checked with the McNemar statistical test. Diagnostic test valuation was calculated with MedCalc software (MedCalc, Osten, Belgium). Sensitivity is defined as probability that a test result will be positive when the disease is present (true positive rate); specificity is probability that a test result will be negative when the disease is not present (true negative rate); positive likelihood ratio (LR+) is ratio between the probability of a positive test result given the presence of the disease and the probability of a positive test result given the absence of the disease; negative likelihood ratio (LR−) is the ratio between the probability of a negative test result given the presence of the disease and the probability of a negative test result given the absence of the disease; positive predictive value (PV+) is probability that the disease is present when the test is positive; negative predictive value (PV−) is probability that the disease is not present when the test is negative and accuracy is defined as overall probability that a patient will be correctly classified. Baseline characteristics were investigated using Fisher’s exact test for categorical and unpaired *t*-test for continuous variables. Statistical analyses were performed using GraphPad Prism 6 and R statistical software. All the analyses with a *p*-value minor or equal to 0.05 were indicated as significant.

## 3. Results

### 3.1. IDH2 R140Q and R172K Detection by PNA-PCR Clamping

**Figure 1:**
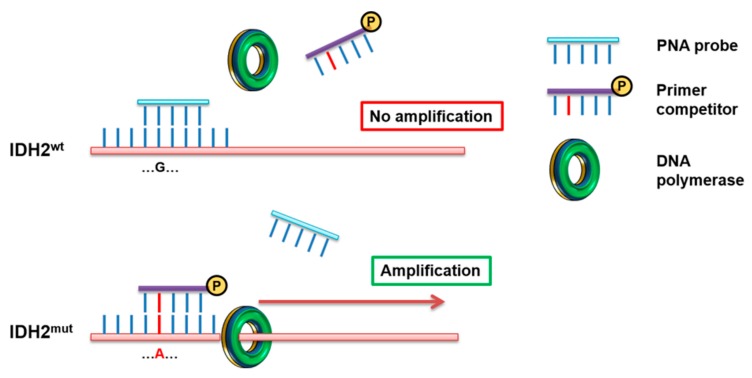
PNA-PCR clamping experimental design. Amplification of *IDH2* was performed in presence of PNA probe (light blue), designed on the WT sequence, and Primer competitor (purple), designed on the mutated sequence. In these conditions, the PCR of the WT sequence is inhibited by the inability of the primer competitor to detach the perfect hybridization PNA/DNA. In contrast, in the presence of *IDH2mut*, PNA/DNA duplex is highly destabilized by the Primer competitor, allowing a strong amplification of the target sequence.

The method described forecasts an overlay between the sequences of PNA probes and the competitor primers *IDH2* forward and *IDH2* R172K reverse (Table 1) respectively for the detection of R140Q and R172K mutations, thus leading to a direct competition towards complementary DNA. In the case of *IDHwt*, a perfect matching occurs between PNA (designed on reference sequence) and DNA, so the PNA-template hybridization is favored, while DNA amplification results suppressed. Contrariwise, a non-perfect matching (as in the case of *IDH2mut*) promotes the hybridization between template and primer competitors, allowing a specific amplification (Figure 1).

**Figure 2:**
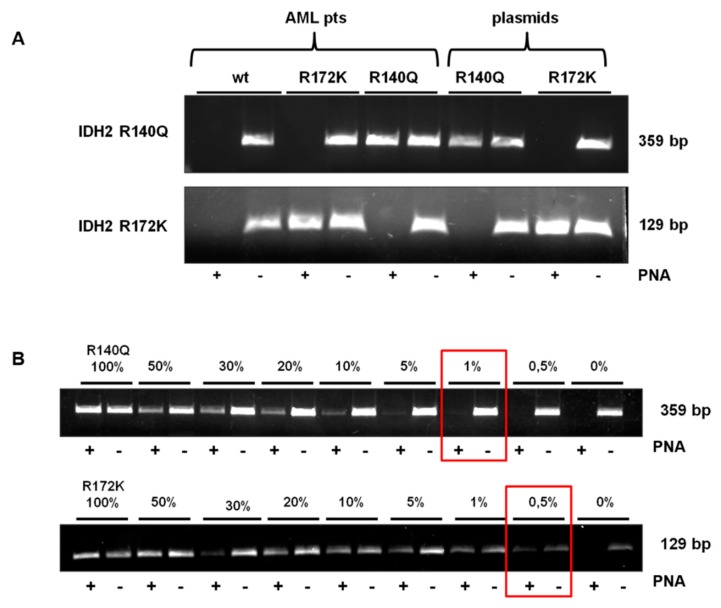
Electrophoretic runs of PCR reactions: 10 μL of each amplicon were loaded on 2% Agarose-TBE 1× gel with 5 μg/mL EtBr and run at 120 V for 30 min. PNA-PCR clamping for *IDH2* R140Q and R172K carried out in absence (PNA−) or in presence (PNA+) of the PNA probe. (**A**) PNA-PCR clamping in DNA from AML samples and control plasmid. The results, read in duplicate, were: *IDH2* WT, if there was no amplification in (PNA+) and amplification in (PNA−) in both the PCR (for R140Q and R172K reaction); *IDH2* R140Q, if there was amplification in both (PNA+) and (PNA−) in R140Q reaction and there was no amplification in (PNA+) and amplification in (PNA−) in R172K reaction; *IDH2* R172K, if there was amplification in both (PNA+) and (PNA−) in R172K reaction and there was no amplification in (PNA+) and amplification in (PNA−) in R140Q reaction. (**B**) PNA-PCR clamping sensitivity assessed mixing at different ratio *IDH2mut* and *IDH2wt* template in the same PCR reaction. Dilutions were as follows: 100, 50, 30, 20, 10, 5, 1 and 0.5% *IDH2mut* (R140Q or R172K) all brought to 100% with the respective amount of *IDH2wt* template. The percentage of the mutated template is indicated. Red boxes highlight the limit of detection of the PNA-PCR clamping method for each mutation in the study.

The amplifications have been carried out in duplicate, in presence (PNA+) or absence (PNA−) of the PNA probes, for both *IDH2* R140Q and R172K reactions. The result was interpreted by reading the double loading for each patient: *IDH2wt* if there was no amplification in (PNA+) and amplification in (PNA−); *IDH2mut* if there was amplification in both (PNA+) and (PNA−) mixtures. Each DNA sample has been screened for both *IDH2* R140Q and R172K with two different PCR (Figure 2A). The detection limit of the assays has been assessed by mixing, at a different ratio, pGEMT-IDH2mut and pGEMT-IDH2wt. Dilutions were as follows: 100, 50, 30, 20, 10, 5, 1 and 0.5% pGEMT-IDH2mut vs. pGEMT-IDH2wt template. The method displayed a low detection limit, allowing us to identify an amount of *IDH2* R140Q template as low as 1% and *IDH2* R172K template as low as 0.5%, which cannot be identified by Sanger sequencing (Figure 2B).

### 3.2. Comparison of Sanger Sequencing, PNA-PCR Clamping and ddPCR for the IDH2mut Detection

**Figure 3:**
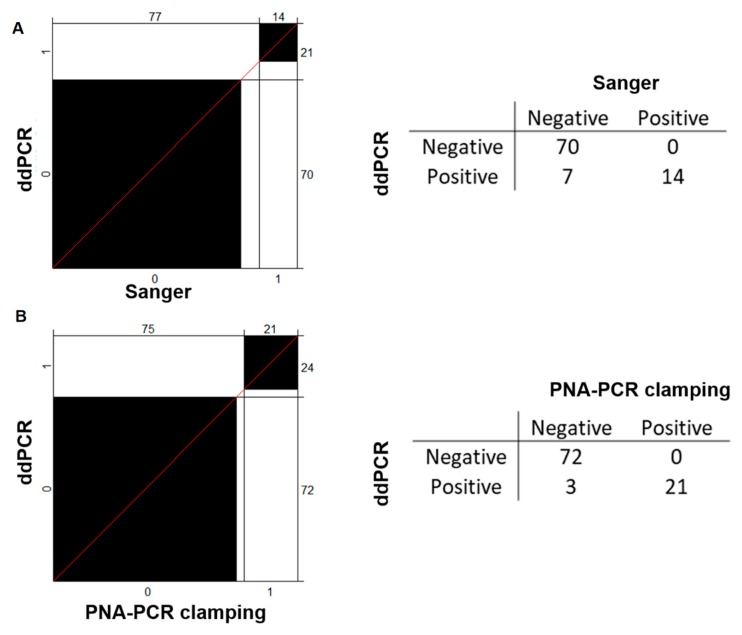
Agreement charts for comparing *IDH2* status evaluation techniques. In agreement plots, the black blocks indicate the accordance of results for each method with respect to the ddPCR, selected as a calibrator for the analysis. ddPCR and PNA-PCR clamping (**B**) had a greater agreement with respect to ddPCR and Sanger sequencing (**A**), as also suggested by the shorter segment of the red 45° diagonal line not included in the black blocks. The 2 × 2 tables alongside underline the differences of each methodology with respect to ddPCR.

Due to the high sensitivity of ddPCR [13], confirmed also in our hands, we considered ddPCR as the reference technique, and we compared the performances of Sanger sequencing and PNA-PCR clamping methods. PNA-PCR clamping, Sanger Sequencing, and the ddPCR were utilized to blind-test 96 DNA from AML patients (74 at diagnosis and 22 during follow-up) for *IDH2* mutations. Since the *IDH2* mutations, R140Q and R172K include more than 95% of all *IDH2* mutations in AML, we subsequently considered the results as a whole, without distinguishing between them. In particular, ddPCR and Sanger identified congruently 70 negative and 14 positive patients. By contrast, seven patients identified as positive by ddPCR were missed in Sanger analysis. Although the two techniques are in agreement in 92.3% of analysis, they are significantly different according to McNemar’s test that checked the disagreements between two matched cases (*p* = 0.02 for Sanger vs. ddPCR) (Figure 3A). As shown in the agreement plot (Figure 3B), PNA clamping and ddPCR were in accordance with 96.9% of the analysis with only three patients differently detected by the PNA clamping test. The agreement between ddPCR and PNA-PCR clamping was confirmed also by McNemar’s test that does not highlight differences in the proportion of disagreement data (*p* > 0.05).

**Figure 4:**
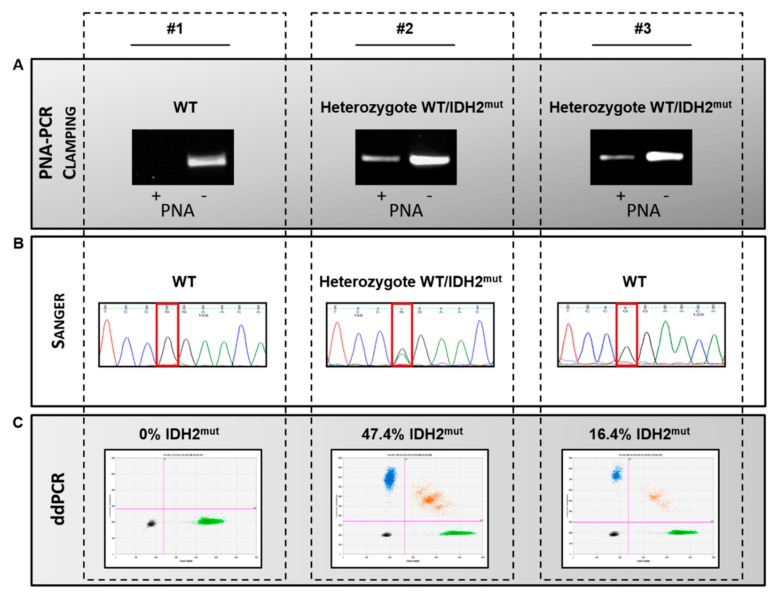
Comparison of techniques for IDH2 status evaluation in three patients affected by AML. (**A**). Electrophoretic runs of PNA-PCR clamping carried out in absence (PNA−) or in presence (PNA+) of the PNA probe. The results, read in duplicate, are: *IDH2wt* (#1), no amplification in (+) and amplification in (−); IDH2mut (#2 and #3), amplification in both (+) and (−). (**B**). Sequencing chromatograms: red boxes indicate nucleotide affected by *IDH2* R140Q mutation. Black peak indicates WT nucleotide (G) in #1 and #3; while double peak black and green (G/A) indicate *IDH2* R140Q heterozygous mutation in #2. (**C**). 2D plots of ddPCR analysis. For each plot, the amplitude in channel 1 (*IDH2* R140Q) is represented on the *y*-axis with the amplitude in channel 2 (*IDH2* WT) represented on the *x*-axis. Four clusters are identified as single-positive for *IDH2* mutated (**blue**) and *IDH2* wild type (**green**), double-positive (**orange**) and double-negative (**grey**). The percentages of *IDH2* R140Q/*IDH2* WT are indicated in each 2D plot.

Figure 4 shows an example of three DNA from AML patients tested with the three different techniques: PNA-PCR clamping (Figure 4A), Sanger sequencing (Figure 4B) and ddPCR (Figure 4C). The example shows that all techniques correctly identified *IDH2* status for patient #1 and #2, wild type and mutated in heterozygosis (47.4% of mutation quantified by ddPCR), respectively. By contrast, patient #3 resulted in *IDH2* mutated by ddPCR and PNA-PCR clamping, while Sanger sequencing failed to identify the *IDH2* mutation (16.4% of mutation quantified by ddPCR).

**Figure 5:**
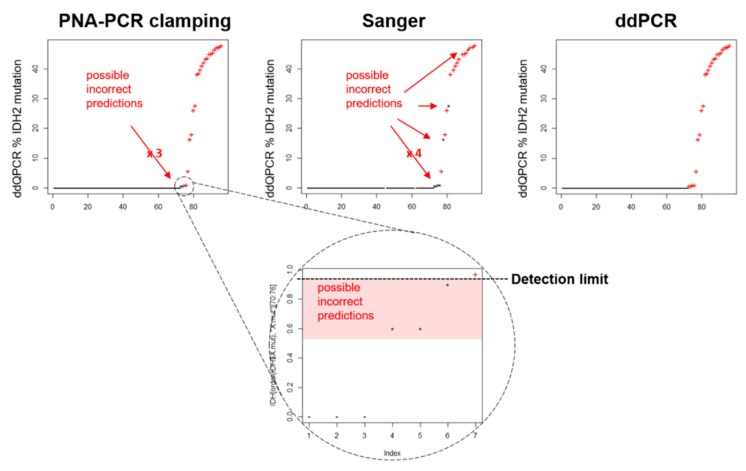
*IDH2* status evaluation techniques detection limits and results agreement. The graphs indicate all the variant allelic frequency (VAF) of *IDH2* mutations identified in AML patients with ddPCR and sorted from 0% (WT) to 50% mutated (heterozygous). ddPCR identified *IDH2* mutant already by 0.6% of VAF, while PNA-PCR clamping failed to identify positive patients under the 1% of VAF (zoom of this region is showed in the dotted circle). Contrariwise, the discriminatory ability of Sanger sequence was randomly distributed in all ranges of IDH2 mutation percentage, decreasing the robustness of the analysis. Negative (−), positive (+) and false negative (*).

These results have brought us to evaluate the detection limit of Sanger and PNA-PCR clamping in patients’ specimens. The results were represented in Figure 5. PNA-PCR clamping showed a linear correlation between the percentage of *IDH2* mutation, evaluated in ddPCR, and the ability to identify the mutation, highlighting a detection limit of about 1%, similar to the detection limit identified by plasmid analysis (Figure 5 magnification). Contrariwise, the discriminatory ability of Sanger sequence was randomly distributed in all ranges of *IDH2* mutation percentage, thus decreasing the robustness of the analysis. Indeed, we evaluated the accuracy of the tests compared to ddPCR, including the sensitivity and specificity, LR+ and LR−, PV+ and PV−. Results indicated that, although specificity and PV− resulted equal for both methods (100%), PNA-PCR clamping was more sensible and accurate than Sanger sequencing (87.5 vs. 66.7% and 96.9 vs. 92.3%, respectively). Further, it showed a better LR− and PV− with respect to the Sanger method (0.12 vs. 0.33 and 96.0 vs. 90.9%, respectively).

### 3.3. Prevalence of IDH2mut in AML Samples

According to previously published data [26], ddPCR detected *IDH2mut* in 12 out of 74 patients at diagnosis (16.2% of the samples), PNA-PCR clamping in 11 (14.8%) and Sanger sequencing in only nine (12.1%). We compared the baseline characteristics of patients carrying the *IDH2mut* and *IDH2wt* patients. We did not find any significant correlation between *IDH2mut* and age, sex, molecular lesions, and *WT1* expression at diagnosis. In accordance with published data, the *IDH2* R140Q and R172K mutations were significantly associated with a normal karyotype (*p* = 0.04) [27]. In particular, only one out of 12 mutated patients showed *MLL* rearrangement between exon 10 and exon 2. Three patients were treated with Enasidenib, two of them became negative for *IDH2* mutation by PNA-PCR clamping and with 0.6% of residual mutation by ddPCR, one remained positive by both methods (43% by ddPCR). One patient was treated with chemotherapy and the mutation was detectable during follow-up by PNA-PCR clamping and ddPCR (from 45% at diagnosis to 38.5 after induction chemotherapy). Finally, one patient was treated with azacitidine and remained stable from 45% to 47% by ddPCR.

## 4. Discussion

AML is a heterogeneous hematological disease, characterized by several karyotypic and molecular alterations. Among these, mutations in *IDH2* gene are found in 8–19% of AML patients [26]. The prognostic significance of *IDH2* mutation at diagnosis is not completely clear and even less clear is the persistence of *IDH2* mutation after chemotherapy, therefore the role of *IDH2* mutation as a marker of MRD needs investigation [12]. In clinical practice, the evaluation of *IDH2* mutations allows a targeted therapeutic choice, based on a specific *IDH2* selective inhibitor (Enasidenib) [28,29]. Frequently the mutated clones at diagnosis are small with a variant allele frequency (VAF) less than 20%, the limit of detection of Sanger sequencing. Furthermore, the identification of residual clones after therapy requires highly sensitive techniques. In this study, we compared a new technology that is sensible, fast, and cheap to identify and monitor *IDH2* mutations based on PNA-PCR clamping and we compared this technique with droplet digital PCR and Sanger sequencing. In our study, PNA-PCR clamping rapidly identified *IDH2* R140Q and R172K mutations in DNA samples with results comparable to ddPCR. In the last years, the PNA-PCR clamping technique showed high efficiency, sensitivity, and specificity and its results were faster and cheaper compared with Sanger sequencing [17,18,19]. We evaluated the *IDH2* status in 96 DNA samples from AML patients with the three methods and we observed that PNA-PCR clamping was in accordance with ddPCR results, in contrast, there was a significant disagreement between Sanger sequencing and the ddPCR results. PNA-PCR clamping showed a limit of detection of about 1% of mutations that was extrapolated in vitro by serial dilutions of mutated plasmids and subsequently confirmed in DNA samples from AML patients. This limit of detection is markedly lower compared to the Sanger method, estimated in the literature to be 15–20% [25]. Further, in our analysis, Sanger sequencing showed some cases of false-negative not correlated with VAF. These sequencing results were probably affected by several external conditions, for example, the quality of DNA. Interestingly, in the same samples, the mutations were detected by PNA-PCR clamping in accordance with ddPCR. This data suggested that PNA-PCR clamping could be an effective method to monitor *IDH2* mutations especially during follow up, where the allele frequency is often below the limit of detection of Sanger sequencing.

Possible disadvantages of our technique could be represented by the fact that PNA-PCR clamping allows only a targeted analysis thus identifying specific mutations defined a priori and it is not able to distinguish a mixed population that often characterizes leukemia. However, these are also limits of Sanger sequencing and ddPCR. This limitation could be overcome only by expensive and time-consuming next-generation sequencing. Considering the accuracy and sensitivity of PNA-PCR clamping, close to ddPCR, its rapidity and its cost, it could be used to early diagnose the presence of *IDH2* mutations, allowing patients to begin a targeted therapy in a reasonable time interval.

## 5. Conclusions

In conclusion, considering the relevance of *IDH2* mutation as a diagnostic and prognostic marker, PNA-PCR clamping could be considered a valid alternative to Sanger sequencing in routine tests and to follow MRD. Further, our assay could be used even in laboratories not equipped for sophisticated analyses, resulting in a faster and cheaper method than the Sanger method and ddPCR, allowing a decrease in the cost and time for advances in molecular investigation and to correctly characterize AML *IDH2* mutated patients.

## 6. Patents

The PNA-PCR clamping assay for IDH2 mutations has been patented by VR, JP, DC and by the University of Turin (Italian patent number 102016000042586).

## Figures and Tables

**Table 1:** Primers and PNA probes sequences for PNA-PCR clamping.

Primer/PNA Probe	Sequence 5′-3′
IDH2 forward	CCAATGGAACTATCCAGAACATC
IDH2 R140Q reverse	CTCGTCGGTGTTGTACATGC
IDH2 R172K reverse	TATATCGCCATGGGCGTGCTT
IDH2 R140Q PNA	CTATCCGGAACATCCT
IDH2 R172K PNA	TGGGCGTGCCTGCCAAT
